# The trade-off between graduate student research and teaching: A myth?

**DOI:** 10.1371/journal.pone.0199576

**Published:** 2018-06-25

**Authors:** Erin E. Shortlidge, Sarah L. Eddy

**Affiliations:** 1 Department of Biology, Portland State University, Portland, Oregon, United States of America; 2 Department of Biological Sciences, Florida International University, Miami, Florida, United States of America; Waseda University, JAPAN

## Abstract

Many current faculty believe that teaching effort and research success are inversely correlated. This trade-off has rarely been empirically tested; yet, it still impedes efforts to increase the use of evidence-based teaching (EBT), and implement effective teaching training programs for graduate students, our future faculty. We tested this tradeoff for graduate students using a national sample of life science PhD students. We characterize how increased training in EBT impacts PhD students’ confidence in their preparation for a research career, in communicating their research, and their publication number. PhD students who invested time into EBT did not suffer in confidence in research preparedness, scientific research communication, or in publication number. Instead, overall, the data trend towards a slight synergy between investing in EBT and research preparation. Thus, the tension between developing research and teaching skills may not be salient for today’s graduate students. This work is proof of concept that institutions can incorporate training in EBT into graduate programs without reducing students’ preparedness for a research career. Although some institutions already have graduate teaching programs, increasing these programs at scale, and including training in EBT methods could create a new avenue for accelerating the spread of evidence-based teaching and improved teaching across higher education.

## Introduction

In recent decades, a multitude of organizations have challenged institutions of higher education to increase student retention in science, technology, engineering and mathematics (STEM) [1–3]. One path to achieving this goal is to increase implementation of the teaching practices that research has largely found to be effective for students. These practices are typically student-centered, and may be referred to as “active learning” or, more broadly, “evidence-based” [2, 4–6]. Despite this national push, scant evidence has been found of systemic movement in faculty teaching practices towards evidence-based teaching (EBT), and, instead, many barriers to change have been identified [7–14]. Although it is critical to continue working with current faculty on EBT, a complementary effort to increase the use of EBT by *future* faculty, current graduate students, has the potential to accelerate the adoption of these practices nation-wide.

Some institutions offer training in EBT that graduate students can opt into if they are aware of the options. These opportunities might include short 1-hr workshops, concentrated ‘teaching boot-camps’, and semester long courses [15, 16]. Unfortunately, short trainings seem to have limited effectiveness for changing teaching practices [16–18]. Effective training programs generally require a substantial time commitmment of a semester or more [10, 19–21]. Empirical examples of the impact of more extensive training is relatively sparse, but all suggest the same pattern. In one study, researchers implemented a GTA training focused on leading inquiry labs and found GTAs self-reported improved teaching skills as a result [22]. PhD students in a multi-year fellowship program run by the National Science Foundation reported improvement in their use of student-centered teaching [21, 23]. Similarly, a year-long program focused on effective teaching practices found 68% of participants reported improvement through the program [24]. Connolly et al. recently found graduate students that engaged in extended training, especially formal courses, described increased self-efficacy in course planning and teaching methods over other forms of teaching development [25]. Thus, the existing studies suggest these longer training programs in EBT can impact at least self-reported use of teaching practices by graduate students.

Currently, many STEM departments do not structure substantial teaching development opportunities for graduate students into their graduate curriculum [24]. If departments do have mandatory trainings, they tend to focus on procedures and policies rather than pedagogy, let alone EBT methods [15, 17, 19, 26–29]. Departments may not embed longer-term pedagogical training into their doctoral programs because of a pervasive perception of a tension between research and teaching in the sciences [19]. This belief is characterized by the assumption that any time spent teaching, or even learning about teaching methods, will take away from research productivity for faculty [14, 17, 30, 31] and graduate students [30, 32]. Research is prioritized in this trade-off, and this prioritization is reinforced by institutional incentivization—research brings in grant money, publications, and prestige that are all emphasized in the promotion and tenure process over teaching. Thus, research is commonly viewed as where more time *should* be spent [17, 32–34].

Although pervasive, this trade-off has rarely been empirically evaluated, and where it has been, the results are mixed. Meta-analyses of the existing research investigating trade-offs between research and teaching found no consistent pattern—at least for faculty [31, 35]. Yet, despite this lack the evidence, the perception of a tension between how academics *should* be spending their time persists [14, 31, 35–43], and is likely passed down to graduate students through their advisor and/or departmental culture [19, 32].

This perception of a tension between research and teaching has at least two potential costs. The first is that by not offering graduate students training in EBT, the spread of EBT practices is slowed. The second is a potential cost to the students themselves. In the United States alone thousands of graduate students earn doctorate degrees each year. In 2016, of these newly minted PhDs, 45% of them where employed in academia [44], indicating an intent to pursue a career in academia which will likely have a teaching component. Even PhDs who ultimately seek a tenure-track position at research-intensive intuitions find themselves in jobs that involve much more than just being proficient at research [32, 37, 45–48]. This point is highlighted in the oft-cited *The Priorities of the Professoriate* report [37], which called for a characterization of the role of faculty to encompass a more holistic vision of scholarship among academic faculty, which includes excellence in teaching. Focusing graduate student training exclusively on research may underprepare these future faculty for the reality of the complex roles faculty members assume, including spending a significant amount of teaching. This under-preparedness is already being felt by some graduate students as illustrated by biomedical postdoctoral scholars reporting that their graduate programs did not provide them with adequate career training and support [49].

Thus, the potential costs of not training graduate students in evidence-based teaching are clear, but is there a trade-off for incorporating teaching development programs into graduate training? Does it hurt their preparedness for a research career? Little work has been done to quantify how graduate students actually invest their time and the impacts of these investments on their progress. One study empirically tested how time spent as a Graduate Teaching Assistant (GTA) related to a student’s ability to write a research grant proposal, and found that students who spent time teaching actually had improved proposal writing skills over those that did not teach [50]. Yet, this study tested impacts of time spent on teaching, not the amount of time spent on learning to teach well.

In the current study, we explore the hypothesis that there is a trade-off between PhD students investing in EBT and being prepared for a research career in academia. We use the term ‘invest’ in EBT because time is consumed when seeking out and gaining training in EBT, and this time is often separate and in addition to time spent as a GTA. We focus on life science PhD students as the life sciences consistently award the greatest number of PhDs across the STEM disciplines—in 2016 23% of all earned doctorates were in the life sciences [44]. We test for a potential trade-off between research and teaching for life science PhD students by investigating three interrelated proxies for preparedness for a research career: confidence that their PhD training program has prepared them to be a researcher, confidence in their ability to communicate their research, and the number of peer-reviewed publications students have produced to date from their PhD work. We chose these three indicators because they have repercussions for retention in research careers in academia. Confidence in one’s research and communication skills are critical components of a successful research career [37, 51–54]. The number of publications a student has out of their PhD is also a significant predictor of long term success in academia [55, 56]. Following the pervasive notion of an antagonism between research and teaching, one could assume that if PhD students invest in EBT training, they will not be as prepared for an academic research career as those who focus less time on training in teaching, and more on research. Here we test for this potential trade-off.

## Materials and methods

### Life Sciences Graduate Student Survey

For this study, life science PhD students were recruited online to complete the Life Sciences Graduate Student Survey, (LSGSS, S1 Fig). The LSGSS was designed to gauge graduate students’ self-reported awareness of, training in, and use of evidence-based teaching methods, as well as report their confidence and training in a variety of tasks and experiences one might have as a graduate student in the life sciences related to research, teaching, and communication. The LSGSS underwent iterative validity measures to establish that the items on the instrument measure the intended constructs [57, 58]. Members of two education research groups as well as multiple STEM graduate students vetted survey items for validity through think-aloud interviews and by piloting the survey and receiving feedback. Based on item feedback, ambiguous questions or unclear wording was addressed and modified for the final instrument. Graduate students were not compensated for participating in the survey and their responses were not linked to their name or other identifying information. The study was approved by the Portland State University IRB (#163844).

#### EBT practices index

Participants reported the training they received in eight common EBT practices in their current graduate program on a four point Likert scale ranging from *no training (1)*, *observation only (2)*, *a little training (3)* to *lots of training (4)*. The practices were chosen based on existing surveys that had been used to determine faculty familiarity with EBT [8]. Because of confusion among participants during think-aloud interviews, several of the initial practices were removed. The final set of EBT practices were presented with written definitions for each practice in the survey, and included: case studies, clickers, concept maps, course-based undergraduate research experiences, discussion-based instruction/Socratic method, flipped classroom, problem-based learning and/or inquiry-based learning, process oriented guided inquiry learning (POGIL), learning assistants, and think-pair-share. Finally, before analysis we removed the learning assistant item and the course-based undergraduate research experience. These showed lower levels of training then any of the other items and unlike the other practices, these lower responses could be driven by institutional differences. If an institution did not support course-based undergraduate research experiences, or a learning assistant program than there would be no reason for training to be offered in it.

We summed student responses on the final 8 items to form an index of their training in EBT. Both observation and no training responses were scored as 0, because they did not represent investment in formal training. *A little training* was coded as 1 and *A lot of training* was coded as 2 to indicated the added time investment this might indicate. Thus, a student with a four for training in EBT could have a little training in four practices, a lot of training on two practices, or a little training on two and a lot of training on one. Our index cannot separate these scenarios, but it does tell us that a student with an index value of four likely invested more time in training than a student with a one (a little training in one practice).

The training in EBT index ranged from 0 to 15 (likely representing a lot of training in 7 of the 8 practices and a little training in 1; S1 Fig). The mean in the sample was 3 ± 3.4 (sd) indicating a little training in three practices or a lot of training in one practice and a little training in one other practice.

#### Outcome variables

The outcome measures of interest were three indicators of a PhD student’s preparation for a research career: confidence in their research training, and confidence in ability to communicate their research, and publications out of their PhD to date. Survey items regarding research and science communication preparedness were modified from a survey measuring the career goals and choices of biomedical postdoctoral scholars [49].

Students answered four Likert-type items regarding their confidence that their graduate training has adequately prepared them to pursue a research career including: writing grant proposals, writing peer-reviewed scientific journal articles, running a research lab, and collaborating on research with individuals of other scientific disciplines. Each individual item had four possible responses ranging from ‘not at all’ to ‘definitely’ adequately trained. We summed student response on the individual questions to create an index of confidence in research training. We created an index instead of considering these items part of a scale that could be averaged because we do not expect students to answer the same way on all the individual items. For example, despite both items representing an aspect of research preparation, it is possible a student could report high confidence in their training in grant writing, but not running a research lab. The research confidence index could range from 4 (no confidence in any aspect of research training) to 16 (absolute confidence in all aspects of research training). The mean response on this index was 11.4 ± 2.6 (S2 Fig).

Students answered three Likert-type items about their confidence communicating their research to other scientists in a professional setting, other scientists in an informal setting, and non-scientists in informal settings. These questions had four response options ranging from ‘not at all confident’ to ‘very confident’. As with confidence in research training, we summed students’ scores on these items to create a research communication confidence index. The index could range from 3 to 12. The mean student response was 9.3 ± 1.8 (S2 Fig).

Finally, participants reported how many peer-reviewed research papers they have published to date from their current PhD program (regardless of authorship position) on a scale from 0 to 3 or more publications. Few students reported two publications, so we pooled this level with the 3+ publications category. Thus, the final outcome variable for number of publications had three levels: 0 publications, 1 publication, and two or more publications (S2 Fig)

#### Control variables

To isolate the effect of training in EBT, we included several potential control variables related to a student’s experience in graduate school. Participants reported the percent of financial support for their PhD that has come from being a GTA. This variable serves as a proxy for the proportion of their time spent teaching and thus not working on their research. Students also reported their year in the PhD program and whether or not they had previously earned a Master’s degree. We expect both of these variables to impact confidence and publication number out of their PhD.

#### Participant recruitment

Students were recruited through professional scientific society listservs, departmental listservs, and snowball sampling. The LSGSS was built using the *Qualitrics* online survey platform, and the survey link was available from June 2016 through August 2016. The survey was accessed by over 900 individuals and completed by over 500 individuals. Graduate students self-selected to take the survey (Table 1). Only individuals who consented for their responses to be used for research and completed the entire survey were kept in the data set.

**Table 1:** Participant demographics.

***Race/Ethnicity***		***Year in PhD Program***		***Career Goals***	
Non-URM	83%	2	22%	Research Faculty	30%
URM	8%	3	25%	Non-academic Research	29%
NA	9%	4	18%	Teaching Faculty	22%
		5	19%	Non-Research	12%
***Gender***		6+	17%	Unsure	7%
Female	58%	***University Type***		***Age (years)***	
Male	36%	R1	72%	< 27	38%
NA	4%	R2/R3	19%	27–30	25%
Other	2%	Other or NA	9%	>30	37%

Description of study sample (N = 338 life science PhD students).

#### Description of study sample

Of the survey respondents 437 were our focal sample: PhD students. We further refined our sample by removing individuals who were in the first year of their PhD program (n = 73), as they have not been in their program long enough for learning about EBTs to have an opportunity to impact their research preparation. In addition, we removed anyone who had already earned a PhD in a different discipline as that experience would make them substantially different from students earning a PhD for the first time and they were too few in number to make this a control variable in the model (n = 17). Finally, we removed students who were seeking non-traditional biology PhDs such as biology education and science and society because we believed the relationships between research and learning about EBTs might be different for students pursuing research in traditional fields like microbiology or ecology. Again, the sample was too small (n = 9) to include this variable as a control.

In summary, the analyses reported here include only the 338 PhD students studying traditional life science research topics (i.e. not biology education, philosophy of science, etc.) who had already completed at least one year of their PhD program before taking the survey (Table 1). Overall, 8.2% of participants were underrepresented minorities and 9.2% did not respond to the race/ethnicity question or were undetermined. The majority of participants identified as female (58.3%), 1.8% were non-binary, and 3.6% did not respond to the question about gender. Participants represented a range of years in their graduate school (2^nd^ year: 21.6%; 3^rd^: 25.1%; 4^th^: 17.7%; 5^th^: 18.9%; 6^th^ or higher: 16.6%). The majority of participants attended very high or high research institutions (72%), the remaining attended moderate research institutions/other institutions by Carnegie Classification of Institutions of Higher Education^®^. Graduate students in the final sample represented 19 different sub-disciplines. The dominant three were: ecology (22.1%), cellular/molecular (17.6%), and evolutionary biology (16.2%). Our study sample is statistically indistinguishable from the life science graduate student population in the US [44] in gender, age, and institution type, but our sample consists of less underrepresented minority life science PhD students compared to national proportions (Chi squared = 34.32; p <0.0001).

#### Model selection

We used linear regression models (outcomes: research preparedness and science communication), and the proportional log-odds model (outcome: publications), to characterize the relationships between the outcome variables, the controls (whether or not a student had a Master’s degree, year in their programs, and proportion of financial support coming from teaching), and training in EBT. Model selection with Akaike’s Information Criterion (AIC) was used to identify the subset of these variables that best fit the data. We started by fitting a complex model that included the control variables, training in EBT, as well as three interaction terms: 1) year in program and proportion of financial support coming from teaching, 2) financial support and training in EBT, and 3) year in program and training in EBT. We searched all possible combinations of these variables to identify the best-fit and most parsimonious models for the three outcome variables. All models were fit in R version 3.3.2 [58] using the MuMIn package [59]. Models with a ΔAIC 2 or less are considered equivalent and models with ΔAIC > 10 are poor predictors of the data.

The models presented in the main body of the paper are the final best-fit and most parsimonious models. We report the 95% confidence set of models for each outcome variable in S1 Table. We then report the regression coefficients from the identified best models for each outcome variable in S2 Table.

## Results and discussion

### Investing in EBT does not reduce sense of being well-trained for a research career

**Fig 1:**
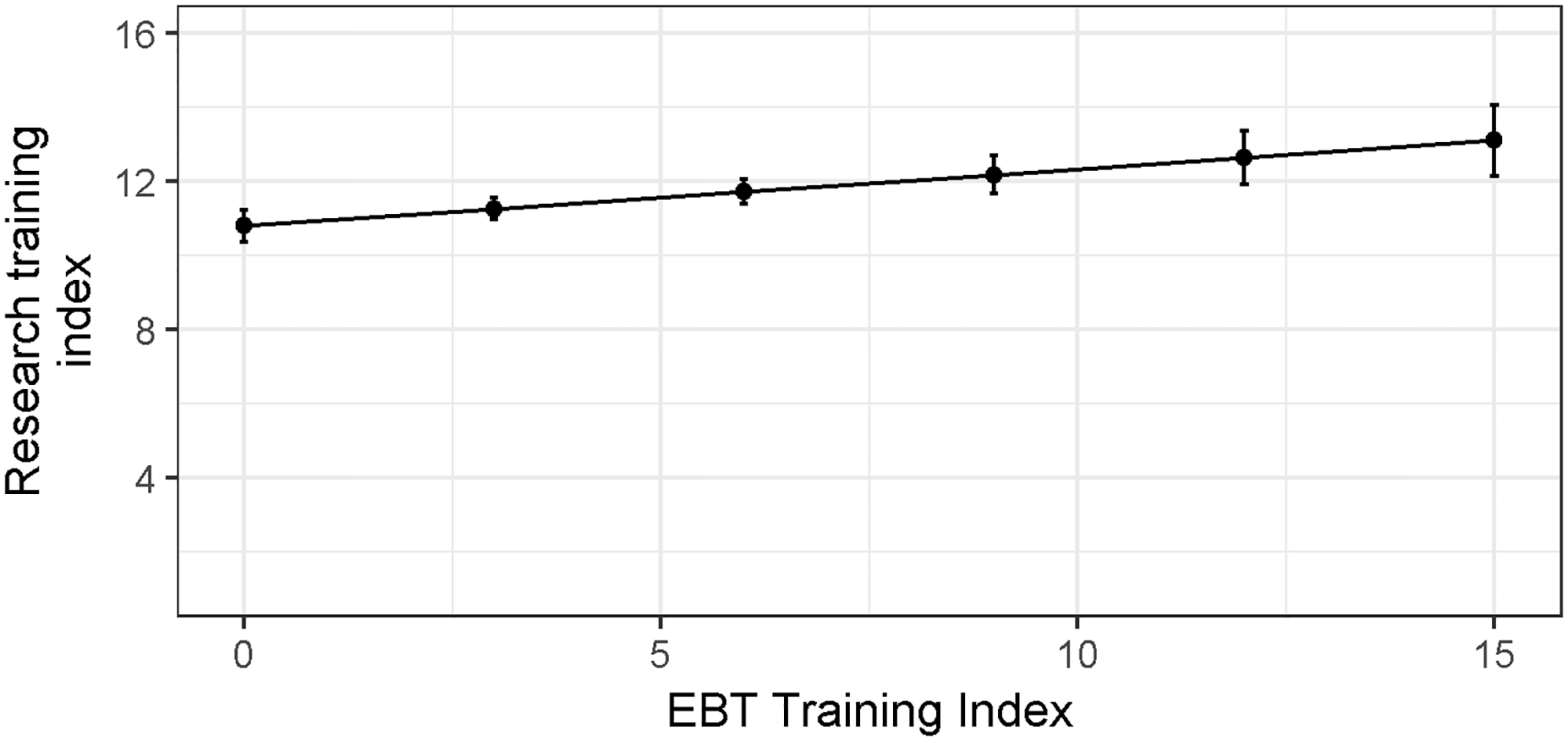
Training in EBT predicts increased confidence in preparedness for a research career. The estimates illustrated in this figure derive from the best-fit linear regression model: Research training adequacy index ~ Training in EBT index (see model selection table S1 Table). Bars represent upper and lower 95% confidence limits around the predicted response.

Increased training in EBT practices did not reduce students’ confidence that their research training has adequately prepared them to be a researcher (Fig 1). Instead, there was a slightly positive relationship (β = 0.15 ± 0.042, t = 3.64, p = 0.0003). Thus, there is no support for the prediction that the time PhD students spend pursuing EBT practices negatively impacts how they perceive their preparedness to do research.

### Investing in EBT does not reduce confidence in ability to communicate research

**Fig 2:**
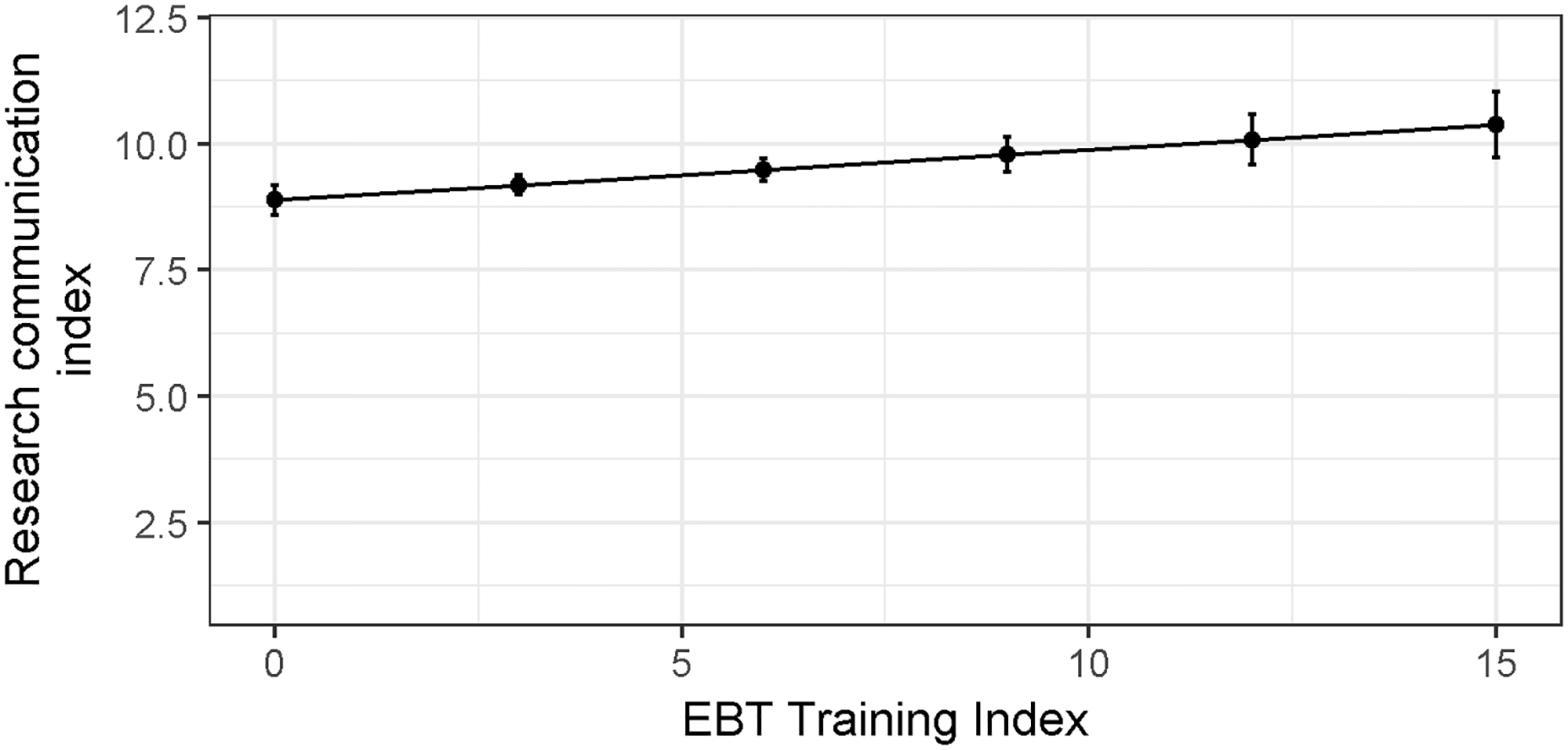
More training in EBT predicts increased confidence in communicating scientific research. The estimates derive from the best-fit linear regression model: Research communication index ~ Training in EBT index (see model selection table S1 Table). Bars represent upper and lower 95% confidence limits around the predicted response.

Student training in EBT increased student confidence in communicating their research (0.10 ± 0.029, t = 3.45, p = 0.0006, Fig 2). Interestingly, the amount of financial support from teaching was not selected to be included in the best models and even in the models it was in the term was not significant. Thus, it seems that teaching experience alone does not increase communication confidence—only training in EBT allows students to reach this desirable outcome.

### Investing in EBT does not reduce publication number

**Fig 3:**
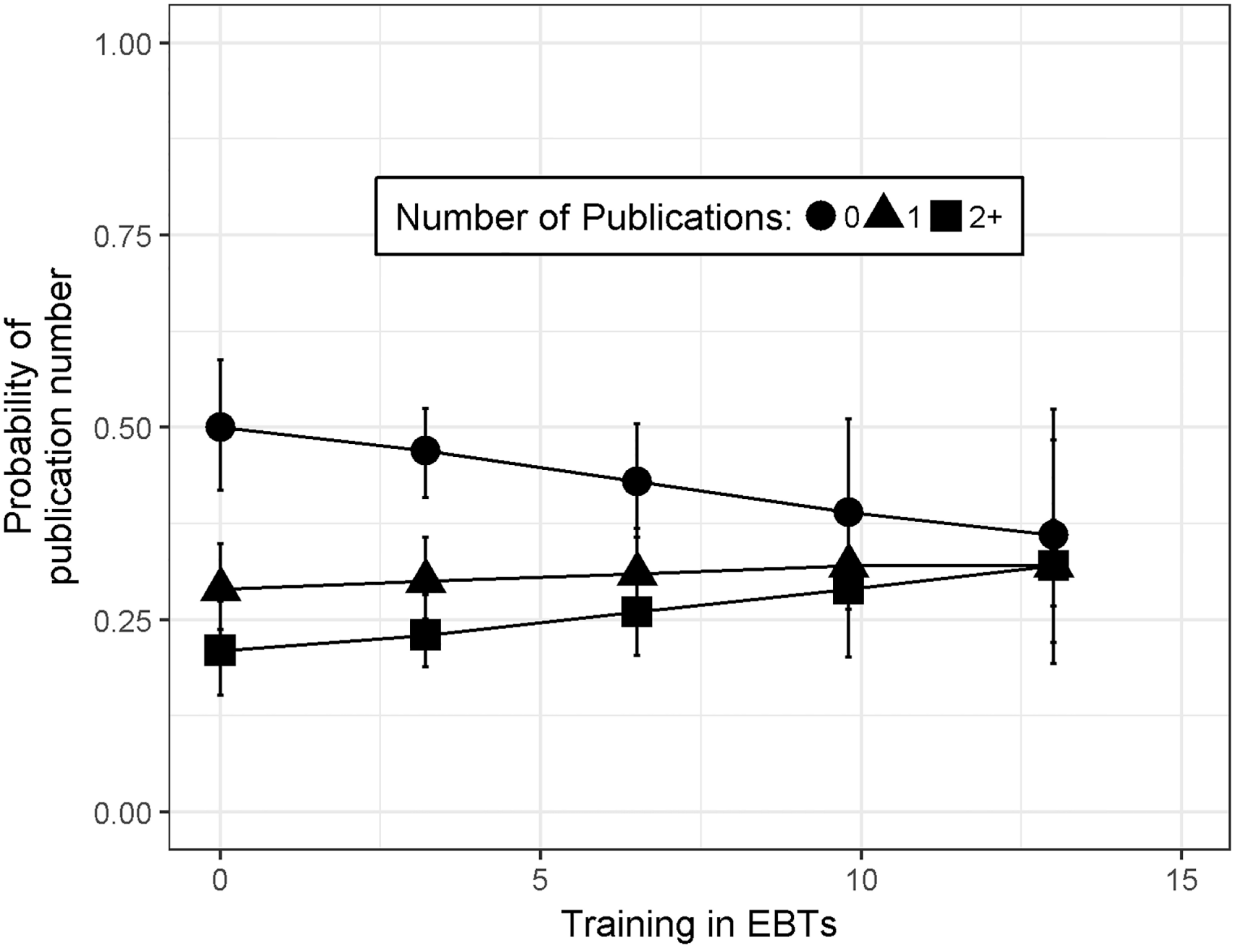
Investment in EBT is not detrimental to research productivity. Graduate student training in EBT does not significantly predict research productivity as measured by number of peer-reviewed publications from their PhD program to date. The estimates illustrated here are derived from the best-fit proportional log odds model controlling for year in program, and whether they already have a Master’s degree. Bars represent upper and lower 95% confidence limits around the predicted probability.

After controlling for whether a PhD student had previously earned a Master’s degree and their year in a PhD program, there was no evidence for a relationship between training in EBT and the number of papers a PhD student had published to date (β = 0.04 ± 0.032, t = 1.32, p = 0.19, Fig 3). These results indicate no support for the hypothesized trade-off between pursuing opportunities to learn about EBT practices during graduate school and a student’s research productivity as measured by published papers. The trend actually hints at the potential for the opposite pattern: for each unit increase in a student’s average training in EBT practices, they were 1.04 times more likely to have at least one additional paper. For example, students with the mean EBT training index had a 47% chance of having zero publications and students in the third quartile of the EBT training index (6.5) were slightly less likely to have zero publications (43% chance).

In higher education, there is widespread perception of an inevitable trade-off between being a productive researcher and investing time in teaching. Academic culture, graduate student mentors, and graduate students themselves may assume that these perceived trade-offs apply to graduate students [17, 19, 45]. Yet, the data presented here do not support this trade-off. Instead, our data support a different hypothesis: graduate students who invest in EBT can be just as competitive of researchers as those who do not make this investment.

Training in EBT practices has been identified as key construct of a recommended framework for GTA training [18], and recent studies suggest that substantial training in EBT can result in the adoption of these practices. A study on the FIRST IV program for post-doctoral scholars demonstrated increased use of EBT after two-years in the professional development program [60]. Similarly, a study found that longer training programs, and especially semester long courses increased graduate student self-efficacy in EBT, and follow-up interviews with these students revealed the majority were employing these methods five years later [19, 25]. These studies, and several studies of teaching self-efficacy (e.g. [25], suggest that investing in these training programs can impact how graduate students teach).

Further, there is beginning to be evidence that graduate students desire this training in EBT. Yet, training in EBT is typically not structured into the PhD training required by their department, and the types of professional development opportunities that result in persistent benefits are not widespread, even at the institutional level [16, 17, 19, 29, 32]. A national study of life science graduate students found that students who had participated in teaching professional development often had to seek out those opportunities beyond their departments [19]. An interview study with a national sample of life science graduate students found similar results and discovered that while most participants had some formal teaching training, only a few claimed receiving substantial training in any student-centered strategies [29]. Yet, encouragingly, 84% of participants saw value in EBT, the majority of which reported having the desire to use them in their own teaching. Together these findings suggest that if institutions invest in providing dedicated programs for graduate students to learn best practices in teaching, they will not be sabotaging graduate student research progress, but will be fulfilling a desire students have to learn the practices and potentially increase implementation of EBT in higher education.

Given our results and those of studies described above, we propose an alternative hypothesis to the ‘trade-off’ between teaching and research preparedness for graduate students: investment in training in EBT may enhance graduate student preparedness for the multifaceted roles that faculty members play [32, 41, 46]. Not providing professional development in pedagogy may undermine a PhD student’s competitiveness relative to students who receive training or have chosen to seek out training independently, especially if the students research productivity and confidence coming out of their PhD programs are otherwise comparable. Given that 38% of graduating PhDs (41% in the life sciences) in 2016 did not yet have definite employment lined up, diversifying one’s portfolio will certainly be key to an individual securing a position in academia, regardless of the specific requirements of that position.

### Limitations

Self-report data has recognized shortcomings including that information can be both under-reported and/or inflated [61] In addition, our sample comes from a self-selecting student population and, despite our sample being fairly representative of the demographics of life science graduate students nationally, it not represent the perceptions or experiences of the overall population of life science graduate students. In particular, in our study the students who engaged in training in EBT likely opted in, so our results may not generalize to students who are required to participate in such training. Yet, the results presented here can be considered proof of concept that training programs in EBT could benefit graduate students rather than harm them.

It is also important to note that we did not directly measure time spent on research, but did include the amount of time supported as a GTA in our models. This would be an important study to do—to understand how EBT training programs impact how and where graduate students invest their time. Finally, this study did not incorporate graduate students from other STEM disciplines. The findings from life science PhD students are a relevant first step to understanding the graduate student experience, and we anticipate broadening the study to other disciplines.

## Conclusions

This research provides initial support for engaging today’s graduate students in EBT—possibly leading to more rapid change in teaching in higher education classrooms. The data demonstrate that PhD students are not hindered by gaining training in EBT in graduate school. If these future faculty move into life science departments valuing research and teaching, they could catalyze a long-awaited paradigm shift towards faculty who develop high quality research programs and simultaneously strive to meet national calls for improved undergraduate education.

## Supporting information

S1 Fig Life Sciences Graduate Student Survey instrument (LSGSS). (DOCX)

S2 Fig Raw distributions of variables used in analyses. Continuous variables are in gray and categorical are in black.
(DOCX)

S3 Fig Raw data. (CSV)

S1 Table 95% confidence intervals of models for the relationships of training in evidence-based practices on three outcomes: Publications from graduate studies, adequacy of training in research, and confidence communicating science. (DOCX)

S2 Table Regression coefficients from best model in the model set for each of the three outcomes with training in EBTS as a predictor. (DOCX)
