# Supplementary material for: The trade-off between graduate student research and teaching: A myth?
Source: PLoS One. 2018 Jun 25;13(6):e0199576. doi: 10.1371/journal.pone.0199576 (PMC6016899; doi:10.1371/journal.pone.0199576)
Supplement: S1 Fig — (DOCX) [file pone.0199576.s001.docx]

**Supporting Information, Shortlidge & Eddy**

**S1 Fig.** Life Sciences Graduate Student Survey Instrument (LSGSS)

Life_Science_Graduate_Student_Experience

Start of Block: Default Question Block

Q1
 **Welcome to the life sciences graduate student survey.**


 **We appreciate your participation!**

| Page Break |  |
| --- | --- |

Q2 Dear Student,
The Biology Education Research Lab at Portland State University is conducting a research study.  
The purpose of the study is to better understand your experience and training as a life sciences graduate student.  You are being invited to participate in this study because you are a life sciences graduate student.   Your participation will involve completing an online survey.  Your involvement in the study is voluntary, and you may choose not to participate.  There are no names or identifying information associated with this survey.  The survey includes questions such as, “Do you feel that your experiences as a life sciences graduate student have or will adequately train you to write grant proposals?”  You can refuse to answer any of the questions at any time.  There are no known risks in this study, but some individuals may experience discomfort when answering questions.  All data will be kept for 2 years in a locked file in the PI's office and then destroyed.   The findings from this project will provide information as to what degree graduate students are aware of evidence-based teaching techniques, and what level of training and experience graduate students have in preparing and employing evidence-based teaching techniques.  If published, results will be presented in summary form only.    If you have any questions about this research project, please feel free to call Erin Shortlidge at (503) 725-9305.  If you have questions regarding your legal rights as a research subject, you may call the PSU Office of Research Integrity at (503) 725-2227.   By completing/returning this survey, you will be agreeing to participate in the above described research study.  You will be provided with a copy of this consent statement.   Thank you for your consideration.  Your participation will help us to better understand the state of life sciences graduate training.   Sincerely,   Dr. Erin Shortlidge Assistant Professor of Biology and Biology Education Portland State University

Q3 **Please select a response:**

- Yes, I wish to participate in the survey. (1)
- No, I do not wish to participate in the survey. (2)

Skip To: End of Survey If Please select a response: = No, I do not wish to participate in the survey.

| Page Break |  |
| --- | --- |

Q4 **Based on your experience as a life sciences graduate student, please indicate your level of confidence with the following:**

|  | Not at all confident (1) | Somewhat confident (2) | Confident (3) | Very confident (4) |
| --- | --- | --- | --- | --- |
| Conducting scientific research related to your research project (1) |  |  |  |  |
| Explaining your research to other scientists in a **formal** setting (e.g., at a scientific conference) (10) |  |  |  |  |
| Explaining your research to other scientists in an **informal** setting (2) |  |  |  |  |
| Explaining your research to non-scientists in an informal setting (3) |  |  |  |  |
| Teaching a science laboratory course as a graduate teaching assistant (TA) (4) |  |  |  |  |
| Teaching a non-laboratory science course as a graduate teaching assistant (TA) (5) |  |  |  |  |
| Mentoring undergraduate students in conducting scientific research (7) |  |  |  |  |
| Mentoring graduate students in conducting scientific research (11) |  |  |  |  |
| Designing science teaching curricula (6) |  |  |  |  |

| Page Break |  |
| --- | --- |

Q5 **Do you feel that your experience as a life sciences graduate student have or will adequately train you to do the following?**

|  | Not at all (1) | Somewhat (2) | For the most part (3) | Definitely (4) |
| --- | --- | --- | --- | --- |
| Write grant proposals (1) |  |  |  |  |
| Write peer-reviewed scientific journal articles (2) |  |  |  |  |
| Communicate science orally (3) |  |  |  |  |
| Obtain a job in industry (4) |  |  |  |  |
| Obtain a job in academia (5) |  |  |  |  |
| Teach your science discipline at the undergraduate level (8) |  |  |  |  |
| Teach your science discipline at the K-12 level (9) |  |  |  |  |
| Teach undergraduate courses outside of your science discipline (13) |  |  |  |  |
| Run your own research lab (10) |  |  |  |  |
| Collaborate with individuals from other disciplines (11) |  |  |  |  |
| Apply your research skills to a field outside of your specialty (12) |  |  |  |  |
| Analyze large data sets (14) |  |  |  |  |
| Use a variety of software programs (15) |  |  |  |  |

| Page Break |  |
| --- | --- |

Q6
**The following series of questions will introduce a number of instructional strategies.  

 We will ask you three questions about each strategy.**
 

| Page Break |  |
| --- | --- |

Q7 Instructional Strategy 1:  
 **Traditional Lecture/Didactic Teaching**:  Instructor acts mostly as a lecturer. Student activity is limited to note-taking and asking occasional questions of the instructor.
 
 
 
Please indicate your level of awareness regarding **Traditional Lecture/Didactic Teaching**based on your experiences as **both** an undergraduate and a graduate student.

- Never heard of it (4)
- Heard of it but do not know much else (2)
- Somewhat familiar with the strategy (3)
- Very familiar with the strategy (5)

Q8 Please indicate the level of training (formal or informal) that you have received **during your graduate program**in using **Traditional Lecture/Didactic Teaching.**

- No training (1)
- Learning by observation only (2)
- Some training (3)
- Lots of training (4)

Q9 Have you integrated **Traditional Lecture/Didactic Teaching** into **your own** formal or informal teaching during your time as a graduate student?

- Yes, I have used Traditional Lecture/Didactic Teaching in my teaching as a graduate student. (1)
- No, I have not used Traditional Lecture/Didactic Teaching in my teaching as a graduate student. (2)
- I have not had formal or informal teaching experience during my time as a graduate student. (3)

| Page Break |  |
| --- | --- |

Q10 Instructional Strategy 2:  
 **Clickers:**Instructors use a classroom response system to collect data from students.
 
 
 
Please indicate your level of awareness regarding **Clickers**based on your experiences as **both** an undergraduate and a graduate student.

- Never heard of it (4)
- Heard of it but do not know much else (2)
- Somewhat familiar with the strategy (3)
- Very familiar with the strategy (5)

Q11 Please indicate the level of training (formal or informal) that you have received **during your graduate program**in using **Clickers.**

- No training (1)
- Learning by observation only (2)
- Some training (3)
- Lots of training (4)

Q12 Have you integrated **Clickers** into **your own** formal or informal teaching during your time as a graduate student?

- Yes, I have used Clickers in my teaching as a graduate student. (1)
- No, I have not used Clickers in my teaching as a graduate student. (2)
- I have not had formal or informal teaching experience during my time as a graduate student. (3)

| Page Break |  |
| --- | --- |

Q13 Instructional Strategy 3:  
 **Concept Maps:**Students diagram the relationships that exist between concepts.
 
 
 
Please indicate your level of awareness regarding **Concept Maps**based on your experiences as **both** an undergraduate and a graduate student.

- Never heard of it (4)
- Heard of it but do not know much else (2)
- Somewhat familiar with the strategy (3)
- Very familiar with the strategy (5)

Q14 Please indicate the level of training (formal or informal) that you have received **during your graduate program** in using **Concept Maps.**

- No training (1)
- Learning by observation only (2)
- Some training (3)
- Lots of training (4)

Q15 Have you integrated **Concept Maps** into **your own** formal or informal teaching during your time as a graduate student?

- Yes, I have used Concept Maps in my teaching as a graduate student. (1)
- No, I have not used Concept Maps in my teaching as a graduate student. (2)
- I have not had formal or informal teaching experience during my time as a graduate student. (3)

| Page Break |  |
| --- | --- |

Q16 Instructional Strategy 4:  
 **Course-based Undergraduate Research Experiences (CUREs):**Students conduct scientific research as part of the course. Students collaborate to collect novel data and produce research that is relevant to the local or broader scientific community.
 
 
 
Please indicate your level of awareness regarding **Course-based Undergraduate Research Experiences (CUREs)**based on your experiences as **both** an undergraduate and a graduate student.

- Never heard of it (4)
- Heard of it but do not know much else (2)
- Somewhat familiar with the strategy (3)
- Very familiar with the strategy (5)

Q17 Please indicate the level of training (formal or informal) that you have received **during your graduate program** in using **Course-based Undergraduate Research Experiences (CUREs).**

- No training (1)
- Learning by observation only (2)
- Some training (3)
- Lots of training (4)

Q18 Have you integrated **Course-based Undergraduate Research Experiences (CUREs)** into **your own** formal or informal teaching during your time as a graduate student?

- Yes, I have used CUREs in my teaching as a graduate student. (1)
- No, I have not used CUREs in my teaching as a graduate student. (2)
- I have not had formal or informal teaching experience during my time as a graduate student. (3)

| Page Break |  |
| --- | --- |

Q19 Instructional Strategy 5:  
 **Discussion-based Instruction/Socratic Method:**Instructor participates in a dialogue with students, or asks them a series of questions in order to guide their examination of the logic and validity of ideas and concepts. 
 
 
 
Please indicate your level of awareness regarding **Discussion-based Instruction/Socratic Method**based on your experiences as **both** an undergraduate and a graduate student.

- Never heard of it (4)
- Heard of it but do not know much else (2)
- Somewhat familiar with the strategy (3)
- Very familiar with the strategy (5)

Q20 Please indicate the level of training (formal or informal) that you have received **during your graduate program** in using **Discussion-based Instruction/Socratic Method.**

- No training (1)
- Learning by observation only (2)
- Some training (3)
- Lots of training (4)

Q21 Have you integrated **Discussion-based Instruction/Socratic Method** into **your own** formal or informal teaching during your time as a graduate student?

- Yes, I have used Discussion-based Instruction/Socratic Method in my teaching as a graduate student. (1)
- No, I have not used Discussion-based Instruction/Socratic Method in my teaching as a graduate student. (2)
- I have not had formal or informal teaching experience during my time as a graduate student. (3)

| Page Break |  |
| --- | --- |

Q22 Instructional Strategy 6:  
 **Flipped Classroom:**Instructor pre-records short lectures for students to access online prior to attending the lecture or classtime.  Classtime is used for activities other than lecture on course content.
 
 
 
Please indicate your level of awareness regarding **Flipped Classrooms**based on your experiences as **both** an undergraduate and a graduate student.

- Never heard of it (4)
- Heard of it but do not know much else (2)
- Somewhat familiar with the strategy (3)
- Very familiar with the strategy (5)

Q23 Please indicate the level of training (formal or informal) that you have received **during your graduate program** in using **Flipped Classrooms.**

- No training (1)
- Learning by observation only (2)
- Some training (3)
- Lots of training (4)

Q24 Have you integrated **Flipped Classrooms** into **your own** formal or informal teaching during your time as a graduate student?

- Yes, I have used Flipped Classrooms in my teaching as a graduate student. (1)
- No, I have not used Flipped Classrooms in my teaching as a graduate student. (2)
- I have not had formal or informal teaching experience during my time as a graduate student. (3)

| Page Break |  |
| --- | --- |

Q25 Instructional Strategy 7:  
**Learning Assistants:**Students who have done well in the course, or in a closely related course, are recruited by the instructor or volunteer to be peer leaders. These peer leaders facilitate small group learning during class time.
 
 
 
Please indicate your level of awareness regarding **Learning Assistants**based on your experiences as **both** an undergraduate and a graduate student.

- Never heard of it (4)
- Heard of it but do not know much else (2)
- Somewhat familiar with the strategy (3)
- Very familiar with the strategy (5)

Q26 Please indicate the level of training (formal or informal) that you have received **during your graduate program** in using **Learning Assistants.**

- No training (1)
- Learning by observation only (2)
- Some training (3)
- Lots of training (4)

Q27 Have you integrated **Learning Assistants** into **your own** formal or informal teaching during your time as a graduate student?

- Yes, I have used Learning Assistants in my teaching as a graduate student. (1)
- No, I have not used Learning Assistants in my teaching as a graduate student. (2)
- I have not had formal or informal teaching experience during my time as a graduate student. (3)

| Page Break |  |
| --- | --- |

Q28 Instructional Strategy 8:  
 **Problem-based Learning (PBL)/Inquiry-based Learning:**Students work in small groups as self-directed teams to solve open-ended problems that require integration of new course material. Instructor acts primarily as a facilitator. 
 
 
 
Please indicate your level of awareness regarding **Problem-based Learning (PBL)/Inquiry-based Learning**based on your experiences as **both** an undergraduate and a graduate student.

- Never heard of it (4)
- Heard of it but do not know much else (2)
- Somewhat familiar with the strategy (3)
- Very familiar with the strategy (5)

Q29 Please indicate the level of training (formal or informal) that you have received **during your graduate program**in using **Problem-based Learning (PBL)/Inquiry-based Learning**

- No training (1)
- Learning by observation only (2)
- Some training (3)
- Lots of training (4)

Q30 Have you integrated **Problem-based Learning (PBL)/Inquiry-based Learning** into **your own** formal or informal teaching during your time as a graduate student?

- Yes, I have used PBL/Inquiry-based Learning in my teaching as a graduate student. (1)
- No, I have not used PBL/Inquiry-based Learning in my teaching as a graduate student. (2)
- I have not had formal or informal teaching experience during my time as a graduate student. (3)

| Page Break |  |
| --- | --- |

Q31 Instructional Strategy 9:  
 **Process Oriented Guided Inquiry (POGIL):**In groups, students work together to complete an assignment designed around a learning cycle of exploration, concept invention and application. Instructor acts primarily as a facilitator.
 
 
 
Please indicate your level of awareness regarding **POGIL**based on your experiences as **both** an undergraduate and a graduate student.

- Never heard of it (4)
- Heard of it but do not know much else (2)
- Somewhat familiar with the strategy (3)
- Very familiar with the strategy (5)

Q32 Please indicate the level of training (formal or informal) that you have received **during your graduate program**in using **POGIL.**

- No training (1)
- Learning by observation only (2)
- Some training (3)
- Lots of training (4)

Q33 Have you integrated **POGIL** into **your own** formal or informal teaching during your time as a graduate student?

- Yes, I have used POGIL in my teaching as a graduate student. (1)
- No, I have not used POGIL in my teaching as a graduate student. (2)
- I have not had formal or informal teaching experience during my time as a graduate student. (3)

| Page Break |  |
| --- | --- |

Q34 Instructional Strategy 10:  
 **Teaching with Case Studies:**Instructor asks students to analyze case studies of historical or hypothetical situations that involve solving problems and/or making decisions.
 
 
 
Please indicate your level of awareness regarding **Teaching with Case Studies**based on your experiences as **both** an undergraduate and a graduate student.

- Never heard of it (4)
- Heard of it but do not know much else (2)
- Somewhat familiar with the strategy (3)
- Very familiar with the strategy (5)

Q35 Please indicate the level of training (formal or informal) that you have received **during your graduate program**in using **Teaching with Case Studies.**

- No training (1)
- Learning by observation only (2)
- Some training (3)
- Lots of training (4)

Q36 Have you integrated **Teaching with Case Studies** into **your own** formal or informal teaching during your time as a graduate student?

- Yes, I have used Teaching with Case Studies in my teaching as a graduate student. (1)
- No, I have not used Teaching with Case Studies in my teaching as a graduate student. (2)
- I have not had formal or informal teaching experience during my time as a graduate student. (3)

| Page Break |  |
| --- | --- |

Q37 Instructional Strategy 11:  
 **Think-Pair-Share:**Instructor poses a problem or question. Students work on the problem individually for a short time, and then form pairs and reconcile their solutions. Pair-work is followed by a whole classroom discussion of students’ responses.
 
 
 
Please indicate your level of awareness regarding **Think-Pair-Share**based on your experiences as **both** an undergraduate and a graduate student.

- Never heard of it (4)
- Heard of it but do not know much else (2)
- Somewhat familiar with the strategy (3)
- Very familiar with the strategy (5)

Q38 Please indicate the level of training (formal or informal) that you have received **during your graduate program**in using **Think-Pair-Share.**

- No training (1)
- Learning by observation only (2)
- Some training (3)
- Lots of training (4)

Q39 Have you integrated **Think-Pair-Share** into **your own** formal or informal teaching during your time as a graduate student?

- Yes, I have used Think-Pair-Share in my teaching as a graduate student. (1)
- No, I have not used Think-Pair-Share in my teaching as a graduate student. (2)
- I have not had formal or informal teaching experience during my time as a graduate student. (3)

| Page Break |  |
| --- | --- |

Q40 Is there an additional instructional strategy, not previously mentioned in this section of the survey, that you would like to bring to our attention?

- Yes (1)
- No (2)

Skip To: Q45 If Is there an additional instructional strategy, not previously mentioned in this section of the su... = No

| Page Break |  |
| --- | --- |

Q41 Additional Instructional Strategy:
 Is there an additional instructional strategy, not previously mentioned in this section of the survey, that you would like to bring to our attention? If so, please describe it here:

________________________________________________________________

________________________________________________________________

________________________________________________________________

________________________________________________________________

________________________________________________________________

Q42 Please indicate your level of awareness about **the additional instructional strategy you described above**based on your experiences as **both** an undergraduate and a graduate student.   *Skip this question if you did not describe an additional strategy.*

- Never heard of it (4)
- Heard of it but do not know much else (2)
- Somewhat familiar with the strategy (3)
- Very familiar with the strategy (5)

Q43 Please indicate the level of training (formal or informal) in **the strategy you described above** that you have received **during your graduate program.**Skip this question if you did not describe an additional strategy.

- No training (1)
- Learning by observation only (2)
- Some training (3)
- Lots of training (4)

Q44 Have you integrated **the strategy you described above** into **your own** formal or informal teaching during your time as a graduate student?   *Skip this question if you did not describe an additional strategy.*

- Yes, I have used this strategy in my teaching as a graduate student. (1)
- No, I have not used this strategy in my teaching as a graduate student. (2)
- I have not had formal or informal teaching experience during my time as a graduate student. (3)

| Page Break |  |
| --- | --- |

| 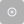 |
| --- |

Q45 Please indicate your level of interest in pursuing each of the following career pathways.

|  | No Interest (2) | Low Interest (3) | Moderate Interest (4) | Interest (5) | Strong Interest (6) | I'm not familiar with this career pathway (1) |
| --- | --- | --- | --- | --- | --- | --- |
| Faculty at a research-intensive institution (1) |  |  |  |  |  |  |
| Faculty at a teaching-intensive institution (2) |  |  |  |  |  |  |
| Faculty at a community college (6) |  |  |  |  |  |  |
| Non-academic research career (e.g., industry, pharmaceutical, biotech, government, start-up) (3) |  |  |  |  |  |  |
| Non-research career (e.g., consulting, policy, science writing, patent law, business) (4) |  |  |  |  |  |  |
| Medical/health career (5) |  |  |  |  |  |  |

Q46 Which career pathway do you **most** hope to pursue after graduation?

- Faculty at a research-intensive institution (1)
- Faculty at a teaching-intensive institution (2)
- Faculty at a community college (8)
- Non-academic research career (e.g., industry, pharmaceutical, biotech, government, start-up) (3)
- Non-research career (e.g., consulting, policy, science writing, patent law, business) (4)
- Medical/health career (5)
- Unsure (6)
- Other (please specify) (7) ________________________________________________

| Page Break |  |
| --- | --- |

Q47 What is your current level of teaching experience? Please select all that apply.

- I have no teaching experience. (1)
- I have informal science teaching experience (e.g., museum or nature park interpreter, outdoor educator). (2)
- I was a learning assistant, workshop leader, or supplemental instruction facilitator as an undergraduate. (10)
- I was a laboratory and/or recitation TA as an undergraduate. (8)
- I have K-12 teaching experience. (3)
- I have been or currently am a laboratory TA in graduate school. (4)
- I have been or currently am a recitation and/or lecture TA in graduate school. (5)
- I have taught lectures when the lead instructor was absent. (6)
- Other (please specify) (7) ________________________________________________

| Page Break |  |
| --- | --- |

Q48 How many peer-reviewed publications have resulted from your graduate research?

- 0 (1)
- 1 (2)
- 2 (3)
- 3 or more publications (4)

Q49 Do you have one or more publication(s) in preparation for submission for peer-review or currently in review? Please only consider publications from your graduate work.

- Yes (1)
- No (2)

| Page Break |  |
| --- | --- |

Q50 Please select the highest degree that you have earned to date.

- Bachelor's degree (1)
- Master's degree (2)
- Professional degree (3)
- Doctorate degree (4)

Q51 From which type of institution did you receive your undergraduate degree?

- Primarily Undergraduate Institution/Small Liberal Arts College (1)
- Comprehensive Institution (Master's Granting) (2)
- Public Research Institution (3)
- Private Research Institution (4)
- Other (5) ________________________________________________

| Page Break |  |
| --- | --- |

Q52 Please name your current graduate institution.

________________________________________________________________

Q53 Please indicate which degree you are pursuing:

- Master's Degree in Arts or Sciences (MA, MS) (1)
- Master's Degree in Science Teaching (MST) (2)
- Other graduate/professional Master's Degree (3)
- Doctor of Philosophy (Ph.D.) (4)
- Other (5) ________________________________________________

Q54 Please choose your current discipline of graduate study:

▼ Agricultural Sciences (1) ... Other (19)

Display This Question:

If Please choose your current discipline of graduate study: = Other

Q55 If you chose "other" for your current discipline of study, please specify below

________________________________________________________________

| Page Break |  |
| --- | --- |

Q56 How many years have you been in your current graduate program?

- 0 - 1 (1)
- 1 - 2 (2)
- 2 - 3 (3)
- 3 - 4 (4)
- 4 - 5 (5)
- 5 - 6 (6)
- 6 - 7 (7)
- More than 7 years (8)

Q57 Please indicate the **total** number of years you anticipate it will take to complete your degree program:

- 2 (1)
- 3 (2)
- 4 (3)
- 5 (4)
- 6 (5)
- 7 (6)
- More than 7 (7)

| 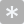 |
| --- |

Q58 To date, what has been the distribution of your financial support for graduate studies? Please **estimate the percentage** for each source of financial support listed below. 
*(Total contributions should equal 100%. If an item is not relevant to your situation, please leave it as "0%.")*

_______ Teaching assistantship (2)

_______ Research assistantship (1)

_______ Fellowship (7)

_______ Other personal earnings, or family earnings, savings or loans (3)

_______ Military or veteran support (14)

_______ Employer reimbursement (6)

_______ Foreign support (4)

_______ Other support (please describe) (5)

| Page Break |  |
| --- | --- |

| 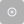 |
| --- |

Q59 With which gender do you identify?

- Decline to state (5)
- Female (2)
- Male (1)
- Transgender (3)
- Other (4) ________________________________________________

| 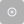 |
| --- |

Q60 Which race/ethnicity best describes you?

- Decline to state (9)
- Black or African American (1)
- Asian/Pacific Islander (2)
- Alaskan Native/American Indian (3)
- Hispanic or Latino/a (4)
- White/Caucasian (5)
- Middle Eastern (6)
- Multiracial and/or Multi-ethnic (7)
- Other (please specify) (8) ________________________________________________

Q61 What is your primary language spoken at home?

- English (1)
- Spanish (2)
- French (3)
- Arabic (4)
- Chinese (Cantonese, Mandarin, or other dialect) (5)
- Japanese (6)
- Other (please specify) (7) ________________________________________________

| Page Break |  |
| --- | --- |

Q62 How long have you lived in the United States?

- 1 year or less (1)
- 2 - 5 years (2)
- 6 - 10 years (3)
- 11 - 19 years (4)
- Over 20 years (5)

Q63 What is your age?

- 22 or under (1)
- 23 - 26 (2)
- 27 - 30 (3)
- 31 and over (4)

Q74 Please name your current graduate institution.

________________________________________________________________

Q75 Please indicate which degree you are pursuing:

- Master's Degree in Arts or Sciences (MA, MS) (1)
- Master's Degree in Science Teaching (MST) (2)
- Other graduate/professional Master's Degree (3)
- Doctor of Philosophy (Ph.D.) (4)
- Other (5) ________________________________________________

Q76 Please choose your current discipline of graduate study:

▼ Agricultural Sciences (1) ... Other (19)

Display This Question:

If Please choose your current discipline of graduate study: = Other

Q77 If you chose "other" for your current discipline of study, please specify below

________________________________________________________________

Q78 How many years have you been in your current graduate program?

- 0 - 1 (1)
- 1 - 2 (2)
- 2 - 3 (3)
- 3 - 4 (4)
- 4 - 5 (5)
- 5 - 6 (6)
- 6 - 7 (7)
- More than 7 years (8)

Q79 Please indicate the **total** number of years you anticipate it will take to complete your degree program:

- 2 (1)
- 3 (2)
- 4 (3)
- 5 (4)
- 6 (5)
- 7 (6)
- More than 7 (7)

| 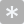 |
| --- |

Q80 To date, what has been the distribution of your financial support for graduate studies? Please **estimate the percentage** for each source of financial support listed below. 
*(Total contributions should equal 100%. If an item is not relevant to your situation, please leave it as "0%.")*

_______ Teaching assistantship (2)

_______ Research assistantship (1)

_______ Fellowship (7)

_______ Other personal earnings, or family earnings, savings or loans (3)

_______ Military or veteran support (14)

_______ Employer reimbursement (6)

_______ Foreign support (4)

_______ Other support (please describe) (5)

| 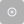 |
| --- |

Q81 With which gender do you identify?

- Decline to state (5)
- Female (2)
- Male (1)
- Transgender (3)
- Other (4) ________________________________________________

| 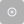 |
| --- |

Q82 Which race/ethnicity best describes you?

- Decline to state (9)
- Black or African American (1)
- Asian/Pacific Islander (2)
- Alaskan Native/American Indian (3)
- Hispanic or Latino/a (4)
- White/Caucasian (5)
- Middle Eastern (6)
- Multiracial and/or Multi-ethnic (7)
- Other (please specify) (8) ________________________________________________

Q83 What is your primary language spoken at home?

- English (1)
- Spanish (2)
- French (3)
- Arabic (4)
- Chinese (Cantonese, Mandarin, or other dialect) (5)
- Japanese (6)
- Other (please specify) (7) ________________________________________________

Q84 How long have you lived in the United States?

- 1 year or less (1)
- 2 - 5 years (2)
- 6 - 10 years (3)
- 11 - 19 years (4)
- Over 20 years (5)

Q85 What is your age?

- 22 or under (1)
- 23 - 26 (2)
- 27 - 30 (3)
- 31 and over (4)

Q86 Please name your current graduate institution.

________________________________________________________________

Q87 Please indicate which degree you are pursuing:

- Master's Degree in Arts or Sciences (MA, MS) (1)
- Master's Degree in Science Teaching (MST) (2)
- Other graduate/professional Master's Degree (3)
- Doctor of Philosophy (Ph.D.) (4)
- Other (5) ________________________________________________

Q88 Please choose your current discipline of graduate study:

▼ Agricultural Sciences (1) ... Other (19)

Display This Question:

If Please choose your current discipline of graduate study: = Other

Q89 If you chose "other" for your current discipline of study, please specify below

________________________________________________________________

Q90 How many years have you been in your current graduate program?

- 0 - 1 (1)
- 1 - 2 (2)
- 2 - 3 (3)
- 3 - 4 (4)
- 4 - 5 (5)
- 5 - 6 (6)
- 6 - 7 (7)
- More than 7 years (8)

Q91 Please indicate the **total** number of years you anticipate it will take to complete your degree program:

- 2 (1)
- 3 (2)
- 4 (3)
- 5 (4)
- 6 (5)
- 7 (6)
- More than 7 (7)

| 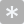 |
| --- |

Q92 To date, what has been the distribution of your financial support for graduate studies? Please **estimate the percentage** for each source of financial support listed below. 
*(Total contributions should equal 100%. If an item is not relevant to your situation, please leave it as "0%.")*

_______ Teaching assistantship (2)

_______ Research assistantship (1)

_______ Fellowship (7)

_______ Other personal earnings, or family earnings, savings or loans (3)

_______ Military or veteran support (14)

_______ Employer reimbursement (6)

_______ Foreign support (4)

_______ Other support (please describe) (5)

| 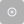 |
| --- |

Q93 With which gender do you identify?

- Decline to state (5)
- Female (2)
- Male (1)
- Transgender (3)
- Other (4) ________________________________________________

| 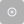 |
| --- |

Q94 Which race/ethnicity best describes you?

- Decline to state (9)
- Black or African American (1)
- Asian/Pacific Islander (2)
- Alaskan Native/American Indian (3)
- Hispanic or Latino/a (4)
- White/Caucasian (5)
- Middle Eastern (6)
- Multiracial and/or Multi-ethnic (7)
- Other (please specify) (8) ________________________________________________

Q95 What is your primary language spoken at home?

- English (1)
- Spanish (2)
- French (3)
- Arabic (4)
- Chinese (Cantonese, Mandarin, or other dialect) (5)
- Japanese (6)
- Other (please specify) (7) ________________________________________________

Q96 How long have you lived in the United States?

- 1 year or less (1)
- 2 - 5 years (2)
- 6 - 10 years (3)
- 11 - 19 years (4)
- Over 20 years (5)

Q97 What is your age?

- 22 or under (1)
- 23 - 26 (2)
- 27 - 30 (3)
- 31 and over (4)

| Page Break |  |
| --- | --- |

Q64 **Are you interested in volunteering to participate in a brief follow-up interview?**
 
If so, please use the link below in order to enter your contact information in a separate survey window. **Your contact information will not be connected to your survey answers.**  

 Your willingness is very much appreciated!
 
https://portlandstate.qualtrics.com//SE/?SID=SV_6hgF2NZE2msLpgF

 Please continue to the next screen to finish the survey.
 
