# Supplementary material for: The trade-off between graduate student research and teaching: A myth?
Source: PLoS One. 2018 Jun 25;13(6):e0199576. doi: 10.1371/journal.pone.0199576 (PMC6016899; doi:10.1371/journal.pone.0199576)
Supplement: S2 Fig — Continuous variables are in gray and categorical are in black. (DOCX) [file pone.0199576.s002.docx]

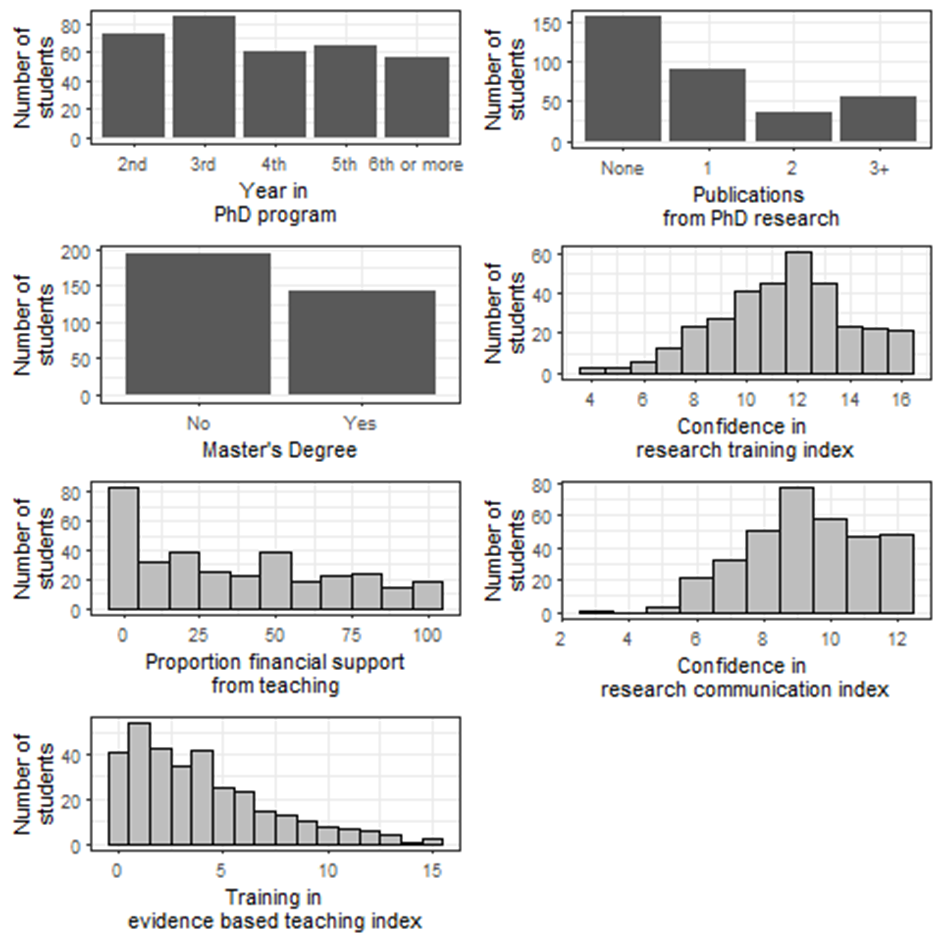


**S2 Fig.** Raw distributions of variables used in analyses. Continuous variables are in gray and categorical are in black.
