# Supplementary material for: The trade-off between graduate student research and teaching: A myth?
Source: PLoS One. 2018 Jun 25;13(6):e0199576. doi: 10.1371/journal.pone.0199576 (PMC6016899; doi:10.1371/journal.pone.0199576)
Supplement: S1 Table — (DOCX) [file pone.0199576.s004.docx]

**S1 Table**. 95% confidence intervals of models for the relationships of training in evidence-bas

ed practices on three outcomes: publications from graduate studies, adequacy of training in research, and confidence communicating science. Models with publications with an outcomes are proportional log odds models and models with research training and communicating science are linear regressions. Models are ranked by AIC. The best model in the model set (with the lowest AIC) is listed first. Only models including training in EBTs were included in the model set. Grayed boxes indicate that the variable was not in the model. White boxes indicate variable was in the model, but it is a factor or is an interaction with a factor, so there is no one number to report. Boxes with numbers indicate the estimate from the model for that continuous variable.

| Outcome: | Training in EBT | Year in Program  (Q56) | Has a Master’s Degree  (Q50) | Proportion of Financial Support from Teaching | Training in EBT x Proportion Financial Support  Teaching | Year in Program  x  Proportion Financial Support  Teaching | Training in EBT  x  Year in Program | AICc |
| --- | --- | --- | --- | --- | --- | --- | --- | --- |
| Publications  (95%  Model Set) | 0.05 |  |  |  |  |  |  | 661.93 |
|  | 0.05 |  |  | -0.002 |  |  |  | 663.67 |
|  | 0.05 |  |  |  |  |  |  | 664.47 |
|  | 0.006 |  |  | -0.006 | 0.001 |  |  | 664.73 |
|  | 0.05 |  |  | -0.002 |  |  |  | 666.33 |
| Adequately Trained in Research (95% Model Set) | 0.17 |  |  |  |  |  |  | 1529.5 |
|  | 0.08 |  |  | -0.01 | 0.002 |  |  | 1530.5 |
|  | 0.17 |  |  | -0.003 |  |  |  | 1531.1 |
|  | 0.16 |  |  |  |  |  |  | 1531.4 |
|  | 0.08 |  |  | -0.01 | 0.002 |  |  | 1532.3 |
|  | 0.17 |  |  | -0.003 |  |  |  | 1532.9 |
| Confidence in Science Communication (95% Model Set) | 0.10 |  |  |  |  |  |  | 1309.9 |
|  | 0.10 |  |  |  |  |  |  | 1310.4 |
|  | 0.10 |  |  | 0.002 |  |  |  | 1311.3 |
|  | 0.10 |  |  | 0.002 |  |  |  | 1312.0 |
|  | 0.13 |  |  | 0.006 | -0.0008 |  |  | 1312.4 |
|  | 0.13 |  |  | 0.005 | -0.0008 |  |  | 1313.0 |
|  | 0.10 |  |  |  |  |  |  | 1315.4 |
|  | 0.09 |  |  |  |  |  |  | 1316.1 |
|  | 0.09 |  |  | 0.003 |  |  |  | 1316.4 |
