# Supplementary material for: The trade-off between graduate student research and teaching: A myth?
Source: PLoS One. 2018 Jun 25;13(6):e0199576. doi: 10.1371/journal.pone.0199576 (PMC6016899; doi:10.1371/journal.pone.0199576)
Supplement: S2 Table — (DOCX) [file pone.0199576.s005.docx]

**S2 Table.** Regression coefficients from best model in the model set for each of the three outcomes with training in EBTs as a predictor. For each model, grayed out boxes indicate that term was not in the best model. A *** after the regression coefficient indicates significance at the 0.001 level, ** indicates significance at the 0.01, and * indicates significance at the 0.05 level.

|  | Outcome:  Publications | Outcome:  Adequately Trained in Research | Outcome:  Confidence in Science Communication |
| --- | --- | --- | --- |
| Intercept |  | 10.8 ± 0.22*** | 8.9 ± 0.15*** |
| Training in EBT | 0.04 ± 0.032 | 0.15 ± 0.042*** | 0.1 ± 0.029*** |
| Year in Program  *(ref: 2^nd^ year)*  3^rd^ year  4^th^ year  5^th^ year  6^th^ or more year | 1.4 ± 0.35***  1.4 ± 0.38***  2.0 ± 0.37***  2.2 ± 0.39*** |  |  |
| Has a Master’s degree  *(ref: No)*  Yes | 0.5 ± 0.22* |  |  |
| Proportion of Financial Support from Teaching |  |  |  |
| Training in EBT  x  Proportion Financial Support Teaching |  |  |  |
| Year in Program  x  Proportion Financial Support Teaching |  |  |  |
| Training in EBT  x  Year in Program |  |  |  |
| R^2^ | NA | 0.04 | 0.04 |
